# Determinants of energy dissipation in the respiratory system during mechanical ventilation

**DOI:** 10.1186/cc14327

**Published:** 2015-03-16

**Authors:** D Massari, C Montaruli, M Gotti, C Chiurazzi, I Algieri, M Amini, M Brioni, C Rovati, A Cammaroto, K Nikolla, M Guanziroli, M Chiodi, A Colombo, M Cressoni, L Gattinoni

**Affiliations:** 1 Università degli Studi di Milano, Milan, Italy

## Introduction

Mechanical ventilation performs at each breath mechanical work on the lung parenchyma. Part of this energy is recovered and part is dissipated into the respiratory system. The purpose of this study is to assess the respective role of three known determinants of energy dissipation: lung opening and closing, strain and lung inhomogeneities [1].

## Methods

Thirteen female piglets (21 ± 2 kg) were ventilated with a strain (V_T_/FRC) greater than 2.5 (V_T_ ~ 38 ± 3 ml/kg) for 54 hours or until massive lung edema developed. Piglets were divided into five groups characterized by different energy loads obtained varying the respiratory rate: 15 breaths/minute (*n *= 3), 12 (*n *= 3), 9 (*n *= 3), 6 (*n *= 2) and 3 (*n *= 2). Every 6 hours two CT scans were performed (end-expiration and end-inspiration) and a static pressure-volume curve was obtained. A total of 51 CT scan couples and 51 corresponding pressure-volume curves was analyzed. The energy dissipated in the lung parenchyma at each breath was determined as the hysteresis area of the pressure- volume curve. CT scans were quantitatively analyzed with dedicated software. Data are presented as median (interquartile range).

## Results

Piglets ventilated with higher energy loads (RR 15 and 12) developed ventilator-induced lung injury (VILI), while piglets ventilated with lower energy loads (RR 9, 6 and 3) did not. The energy dissipated in the lung parenchyma at each breath was 0.56 J (0.52 to 0.62). Dissipated energy increased with time in piglets that developed VILI, while it remained near-constant in all the other piglets. Recruitability was 6% (3 to 8) of lung parenchyma, strain was 3.1 (2.6 to 3.5) and lung inhomogeneity extent (that is, the percentage of lung parenchyma that is inhomogeneous) was 10% (9 to 11). The energy dissipated in the lung parenchyma was well related to lung recruitability (*r*^2 ^= 0.50, *P *< 0.0001), strain (*r*^2 ^= 0.57, *P *< 0.0001) and lung inhomogeneity extent (*r*^2 ^= 0.42, *P *< 0.0001). Multiple linear regression showed that dissipated energy was independently related to all of the three determinants of energy dissipation: lung opening and closing (*P *= 0.025), strain (*P *< 0.0001) and lung inhomogeneities (*P *< 0.01).

## Conclusion

Energy dissipation was largely dependent on strain, obtained with very high tidal volumes, but also lung inhomogeneities and lung opening and closing played a significant role.
